# Phase III, multi-centre, randomised, double blind, Placebo-controlled study for treatment of juvenile ankylosing spondylitis (AS) with Adalimumab

**DOI:** 10.1186/1546-0096-9-S1-P201

**Published:** 2011-09-14

**Authors:** G Horneff, S Fitter, HI Huppertz, I Foeldvari, K Kuemmerle-Deschner, R Kuester, N Tzaribacev, A Thon, M Borte, G Ganser, R Trauzeddel, K Minden

**Affiliations:** 1 Asklepios Clinic, Sankt Augustin, Germany; 2 Prof Hess Children's Hospital, Bremen, Germany; 3 Paediatric Rheumatology Office, Hamburg, Germany; 4 University Tuebingen, Tuebingen, Germany; 5 Centre Paediatric Rheumatology, Bad Bramstedt, Germany; 6 Paediatric Rheumatology Centre, Bad Bramstedt, Germany; 7 MMH Hannover, Hannover, Germany; 8 St. Georgs Hospital, Leipzig, Germany; 9 Paeditric Rheumatology Centre, Sendenhorst, Germany; 10 Helios Clinics, Berlin, Germany; 11 Charite, Berlin, Germany

## Background

TNF-inhibitors are valuable treatment options for adult AS but are not approved for juvenile AS.

## Aim

To determine efficacy of adalimumab (ADA) in juvenile AS in a 12 wks double blind placebo controlled trial.

## Methods

In total 32 subjects aged from 12 to 17 years suffering from juvenile AS were enrolled into the clinical trial HUM06-037. All subjects were randomized and included in the ITT analysis, among them 2 who terminated the treatment prematurely after 3 injections due to insufficient efficacy (one of each group) and were label as non-responders.

## Results

17 patients were randomized to receive ADA 40mg/2wks and 15 patients received placebo. Stable doses of NSAIDs and low dose of corticosteroids (≤10 mg per day) were permitted. In the ADA group a decrease of all disease activity parameters was noted at wk 12: BASDAI spinal inflammation score -66% (p<0.001); back pain score -48% (p<0.005); BASFI score -47 % (p<0.02); CHAQ-DI score -65% (p<0.005); ESR -75% (p<0.05) and finally the CRP - 80% (p=0.07).

ASAS20 and 40 response rates after 4, 8 and 12 weeks were higher on ADA than in the PLC group (table 1)

**Table 1:** per protocol analysis

	ASAS20			ASAS40		
Wk.	PLC	ADA	p	PLC	ADA	p
4	4 (31%)	9 (56%)	0.24	3 (23%)	7 (44%)	0.19
8	3 (23%)	10 (63%)	0.03*	3 (23%)	9 (56%)	0.07
12	4 (31%)	9 (63%)	0.17	4 (31)	9 (56%)	0.17

During the 12 wks controlled phase 26 AEs occurred in 10 pts. on PLC compared to 25 AEs in 11 pts on ADA. Injection site reactions were the most common adverse event (10 on PLC, 11 on ADA). There were 16 various infections occurring in the double blind phase, 6 on PLC, 10 on ADA. 2 SAEs occurred on ADA.

## Conclusions

This small placebo controlled trial demonstrated significant effects of adalimumab treatment in patients with juvenile ankylosing spondylitis.

